# Din Oversees Mesenchymal Stem Cell Homeostasis in Mouse Incisors

**DOI:** 10.21203/rs.3.rs-6568233/v1

**Published:** 2025-05-14

**Authors:** Xiaofang Wang, Changchun Dong, Bikash Lamichhane, Sanjaya Thapa, Yongxu Zhang, Shreyan Gupta, James J. Cai

**Affiliations:** Texas A&M University College of Dentistry; Texas A&M University College of Dentistry; National Institute of Dental & Craniofacial Research; Texas A&M University College of Dentistry; Texas A&M University College of Dentistry; Texas A&M University; Texas A&M University

## Abstract

The murine incisor presents an excellent model for investigating stem cell homeostasis due to its regenerative capacity and continuous growth throughout the lifetime. Proper homeostasis of the dental epithelial stem cells (ESCs) and mesenchymal stem cells (MSCs) is pivotal for the continuous growth, tissue turnover and injury healing in murine incisors. By employing a newly developed knockout mouse model, we revealed that a predicted gene, *Din* (*4930453N24Rik*), plays pivotal roles in the homeostasis of MSCs in murine incisors. *Din*-deficient incisors exhibited arrested growth after eruption, and severely compromised healing/regeneration ability following injury. Although *Din* showed expression in multiple cell types in murine incisors, including both dental epithelium- and dental mesenchyme-derived naïve and differentiated cells, lineage-specific knockout of *Din* from epithelium, cranial neural crest, *Col1a1*-expressing cells, and *Gli1*+ MSCs indicated that *Din* is essential for the dental MSCs in murine incisors but dispensable for the dental ESCs and differentiated ameloblasts and odontoblasts. Single-cell RNA sequencing (scRNA-seq) analysis revealed a decline in *Din* expression levels along the MSC differentiation trajectory, with highest levels in MSCs and transit amplifying cells (TACs), followed by low levels in pulp fibroblasts and odontoblasts. *Din*-deficient MSCs exhibited receded stemness, reduced motility, accelerated aging, and compromised osteogenesis potential whilst enhanced adipogenesis potential. Our transcriptomic, proteomic, and GLISA assays collectively suggest that Din may oversee multiple aspects of MSC homeostasis in murine incisors through Rho GTPases.

## INTRODUCTION

The mutation of *Din* gene arose in the Jackson Laboratory inbred C3H/HeJ strain mice in 1984^1^. The incisors of the mutant mice showed phenotypes in an autosomal recessive trait, including arrested growth after eruption, “overgrowth” of dentin, and a reduced pulp chamber, collectively referred to as Dense incisors or Din^1^. The causal gene for Din was initially mapped to chromosome 16 by linkage analyses^1^, and later identified as the predicted gene *4930453N24Rik* via whole exome sequencing^2^. So far, little is known about the function of *Din* gene. The “overgrowth” of dentin due to arrested growth of incisors in *Din* mice, reminiscent of similar phenotypes observed in several mutant mice with dysregulated signaling in incisor stem cells or transit amplifying cells (TACs)^3–5^, which implies a potential role of *Din* in the homeostasis of murine incisors, particularly considering that mice experiencing issues with incisor renewal often exhibit similar phenotypes^3–7^.

Mice possess the unique ability to completely renew their incisors every 35 to 45 days. This trait, shared by all rodents, makes mice highly valuable for studying the regeneration of tissues through stem cells. Within a month, the epithelial and mesenchymal compartments of murine incisors replenish all their cells rapidly^8^. The self-renewal of incisor relies on quiescent epithelial stem cells (ESCs) in the cervical loop region^9, 10^ and mesenchymal stem cells (MSCs) localized near the cervical loops and neurovascular bundle that give rise to TACs^11, 12^. The ESCs and MSCs at the proximal side of incisor supply the progenitors that differentiate into ameloblasts and odontoblasts to form enamel and dentin continuously^3, 6, 13^. In response to injury or stress, the repair system of murine incisors gets activated which are backed by these stem cells that have intrinsic ability to self-renew and regenerate tissue-specific mature cells to replace damaged enamel, dentin and dental pulp. The tissue turnover and healing process require the continuous supply of cells that can divide over a period of time. Thus, the homeostasis of stem cells and their differentiation into the required tissue-specific cells are a must for proper turnover/healing which are governed by various signaling mechanisms^14^.

Stem cell homeostasis involves a series of tightly regulated cellular events, including population maintenance, cell cycle switching, directed migration, and guided differentiation, etc. Proper homeostasis of stem cells is crucial for postnatal growth, tissue turnover, and injury repair in both human and rodent teeth^3, 15^. Several regulatory mechanisms have been identified associated with the homeostasis of ESCs during enamel renewal/turnover in murine incisors^9, 16–19^. Due to the significant application prospects of MSCs in regenerative medicine/dentistry, there is a greater focus on research regarding MSC homeostasis in murine incisors, which is associated with tissue turnover and injury healing in dentin-pulp complex^3–5, 7, 12, 20–22^. These studies have revealed the regulatory mechanisms governing specific aspects of MSC homeostasis in mouse incisors. However, it remains unclear whether there are factors overseeing various aspects of MSC homeostasis and if a cohesive regulatory mechanism exists to coordinate the processes.

Small GTPases function as nodal points that integrate upstream regulatory inputs and disseminate effector outputs to modulate extensive signaling pathways and cytoskeleton reorganization to regulate cell proliferation, differentiation, polarization and motility, etc. They make binary on/off decisions through controlled loading of GTP (activation) and hydrolysis of GTP to GDP (inactivation). The cellular regulation of this cycle involves guanine nucleotide exchange factors (GEFs) which accelerate intrinsic GDP/GTP exchange, GTPase-activating proteins (GAPs) which **terminate the signaling event**, and guanine nucleotide dissociation inhibitors (GDIs) which bind to prenylated GTPases and control their cycle between the cytosol and membrane^23^.

It has been shown that small GTPases regulate the homeostasis and cell fate of several stem cell lineages^24–34^, and are implicated in tooth development and tissue turnover. For instance, integrin α3 regulates TAC proliferation via FAK and Cdc42, driving tissue renewal in mouse incisors^35^; RhoA, Rac1, and ROCK are associated with ameloblast differentiation^36^; and CDC42-mediated Wnt signaling promotes odontogenic differentiation of dental papilla cells during tooth root elongation^37^.

In this study, we demonstrate that *Din* is essential for MSC homeostasis in murine incisors, but is dispensable for odontoblasts and dental epithelium-derived cells, such as ameloblasts and ESCs. *Din* oversees multiple aspects of MSC homeostasis, including stemness, proliferation, differentiation, migration, and population maintenance. These functions are likely mediated through the binary molecular switches of Rho GTPases.

## RESULTS

### Din is Essential for the Postnatal Growth and Renewal of Murine Incisors.

**Figure 1:**
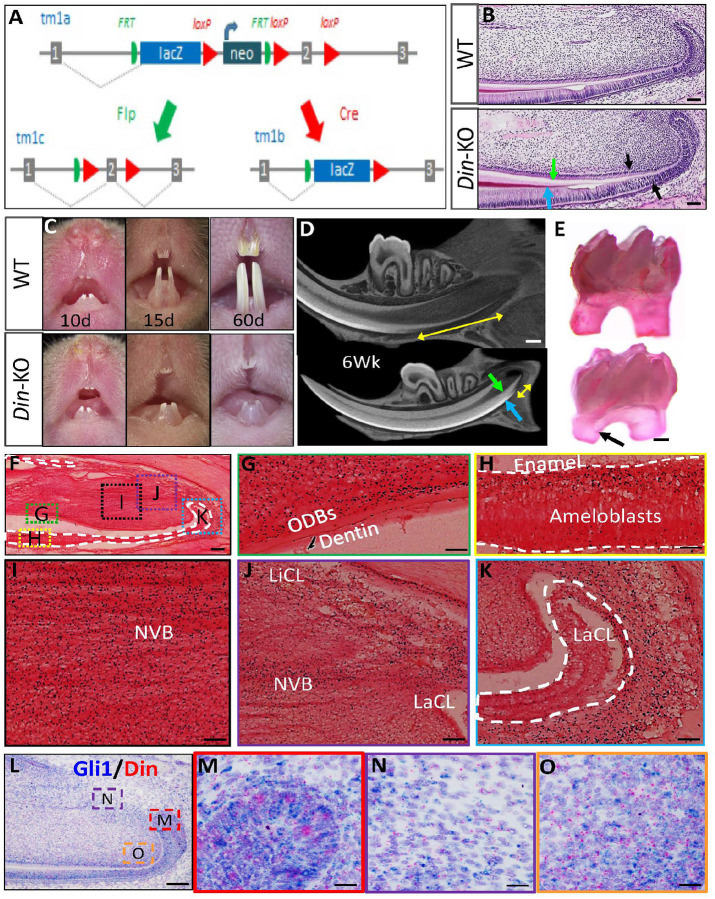
Generation of *Din*-mutant alleles, tooth phenotypes in *Din*-KO mice, and *Din* expression pattern in mouse lower incisors. **A.** Generation of *Din*-KO and *Din*-flox alleles. tm1a, gene-trap allele targeting intron 1 and exon 2 of *Din* gene. tm1b, *Din*-KO allele was generated by crossbreeding the gene-trap allele tm1a with a germline-expressing *Cre* allele to remove Neo cassette and exon 2. tm1c, *Din*-flox allele was generated by crossbreeding the gene-trap allele tm1a with a germline-expressing *Flp* allele to remove LacZ and Neo cassettes. **B.** HE staining of sagittal sections of lower incisors from P10 mice revealed more mature and higher column morphology of pre-ameloblasts and pre-odontoblasts in *Din*-KO mice (black arrows) compared to WT. Accordingly, the dentin (green arrow) and enamel matrix (blue arrow) in *Din*-KO incisors showed an early onset of secretion. **C.** Front view of upper and lower incisors in *Din*-KO and WT mice at postnatal ages 10, 15, and 60 days. The *Din*-KO incisors showed arrested growth after eruption. **D.**Sagittal sections of 3D reconstructed μ-CT images of lower incisors of 6-week-old mice. The *Din*-KO incisors showed “overgrown” dentin and obstructed pulp chamber. Mature dentin (green arrow) and enamel (blue arrow) extended to the proximal side near cervical loops. The distance of proximal region without mature dentin and enamel (double-arrowed yellow line) was dramatically reduced in *Din*-KO incisors. **E.** The molars of *Din*-KO mice showed nearly normal crown and underdeveloped roots. **F.** X-galactosidase staining on sagittal cryosections of lower incisors from 4-week-old *Din*-KO heterozygotes (*Din* expression was indicated by LacZ). Counterstained with Nuclear-Fast Red. **G.** Enlarged box G in F. LacZ was detected in pulp cells and odontoblasts (ODBS). **H.** Enlarged box H in F. LacZ was detected in ameloblasts. **I.**Enlarged box I in F. The neurovascular bundle (NVB) in dental pulp showed robust LacZ staining. **J.** Enlarged box J in F. The MSC niche between lingual cervical loop (LiCL) and labial cervical loop (LaCL) showed robust LacZ staining. **K.** Enlarged box K in F. The cervical loop barely had *Din* expression, while the dental follicle cells posterior to LaCL showed robust LacZ staining. **L.** Dual RNAScope staining of *Din* and *Gli1* on sagittal FFPE sections of lower incisors from 4-week-old mice. Counterstained with hematoxylin. **M.**Enlarged box M in L. *Din* and *Gli1* showed co-expression in cervical loop cells. **N.** Enlarged box N in L. *Din* and *Gli1* showed co-expression in MSC niche cells. **O.** Enlarged box O in L. *Din* and *Gli1* showed co-expression in mesenchymal TACs. Scale bars: B, 100 μm; D, 1 mm; E, 0.5 mm; F and L, 200 μm; G-K and M-O, 50 μm.

*Din* deficiency did not affect the development of embryonic teeth but significantly compromised the postnatal growth and renewal of murine incisors and root development of molars. Histology of embryonic and postnatal incisors did not identify differences between *Din*-KO and WT until postnatal 10 days (Fig. S1). *Din*-KO incisorscan normally erupt into oral cavity. Subsequently, they started showing extremely slow or arrested growth (Fig. 1B and C), while their dentin continued accumulating until the pulp chamber was obstructed at older age (Fig. 1D, Fig. S2). Mature dentin and enamel were observed at the proximal side near cervical loops where normally they should not be present (Fig. 1D, Fig. S2). The molars of *Din*-KO mice showed a largely normal crown, with compromised postnatal growth of dental roots (Fig. 1E). The presecretory and secretory ameloblasts and odontoblasts in P10 *Din*-KO incisors displayed more mature and higher column morphology than WT (Fig. 1B), and laid down more dentin and enamel matrix at the proximal side near the cervical loops (Fig. 1B and D). No differences were identified in the teeth between male and female *Din*-KO mice.

### The Expression Pattern of Din in Murine Incisors.

The *Din*-KO allele had a LacZ reporter driven by endogenous *Din* promoter, which can be used to indicate *Din* expression (Fig. 1A). X-Gal staining on sagittal cryosections of lower incisors from 4-week-old *Din*-KO heterozygotes (normal mice) showed that *Din* is expressed in both differentiated and undifferentiated dental cells, including pre- and mature odontoblasts and ameloblasts (Fig. 1F–H), and dental pulp cells in the proximal neurovascular bundle (NVB) and between the labial and lingual cervical loops, where the putative dental MSC niches reside (Fig. 1I and J). Labial cervical loop (LaCL) only showed sparse expression, while the dental follicle cells posterior to LaCL displayed robust signals (Fig. 1K).Dual RNAScope staining of *Din* and *Gli1* showed co-expression of the two transcripts in *Gli1*+ MSCs and TACs in murine incisors (Fig. 1L–O).

### Din is Essential for Dentinogenesis but Dispensable for Amelogenesis in Murine Incisors.

**Figure 2:**
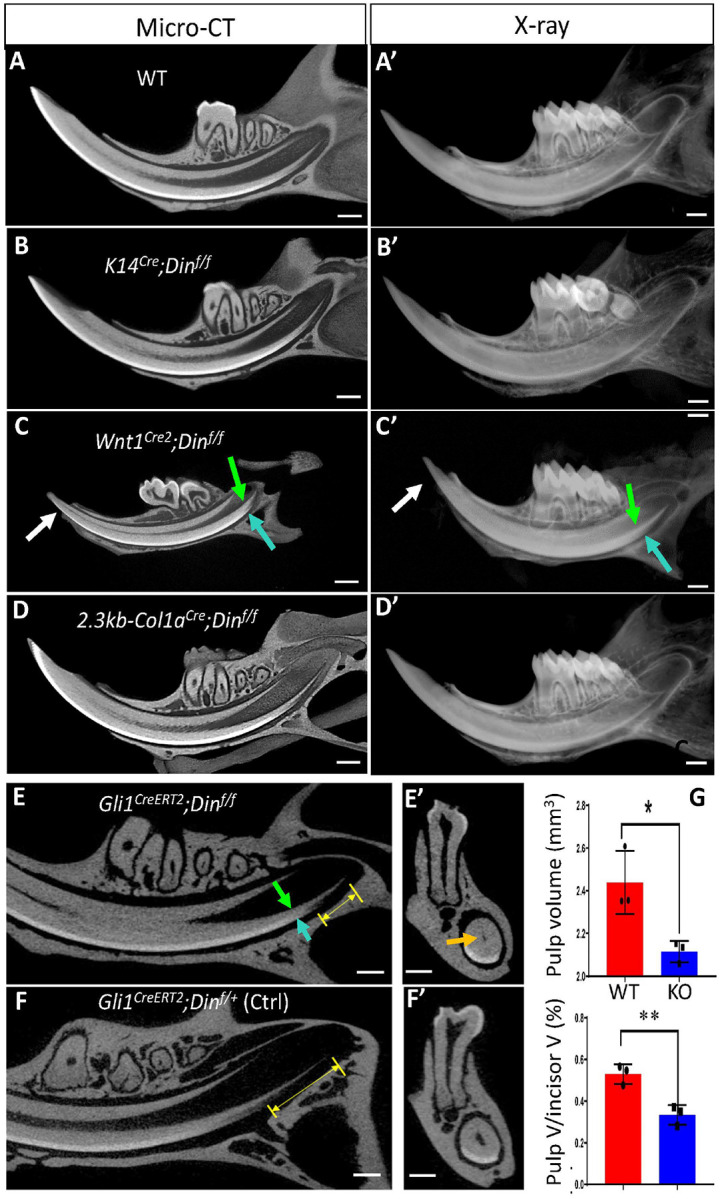
Conditional knockout (cKO) of *Din* from different cell lineages. **A.** Lower incisors of 6-week-old WT mice. **B.** *K14*-Cre derived *Din*-cKO from epithelial cells did not cause any dental abnormalities in lower incisors. **C.** *Wnt1*-Cre2 derived *Din*-cKO from cranial neural crest cells recapitulated the phenotypes of dentin (green arrow), enamel (blue arrow), and arrested growth after eruption (white arrow) observed in *Din*-KO incisors. **D.** 2.3kb *Col1a*-Cre derived *Din*-cKO from *Col1a*-expressing cells did not cause any dental abnormalities in lower incisors. **E.** *Gli1*-CreERT2 derived *Din*-cKO from *Gli1*-expressing MSCs resulted in obstructed pulp chamber (orange arrow), proximal dentin (green arrow) and enamel (blue arrow), and reduced length of immature dentin and enamel in cervical loop region (double-arrowed yellow lines) in the lower incisors of 4-month old cKO mice in comparison with heterozygous control (Ctrl) in **F. G.** The incisor pulp volume and the ratio of pulp/incisor volume were significantly reduced in *Gli1*-CreERT2-derived cKO mice in comparison with control mice. N=3. *, P<0.01. **, P<0.001. Scale bars, 1 mm.

The development of dentin and enamel involves reciprocal interactions between the dental epithelium and dental mesenchyme. Given that *Din* expression was detected in cells derived from both dental epithelium and dental mesenchyme, and that the dental defects observed in *Din*-KO mice incisors affected both dentin and enamel, there is a need to clarify whether *Din* functions in either dental tissue or both of them. To this end, *Din-floxed* mice were crossbred with *K14-Cre* and *Wnt1-Cre2* transgenic mice to conditionally knock out (cKO) *Din* from either tissue. Surprisingly, the *K14-Cre* derived cKO mice did not show any dental abnormities, while the *Wnt1-Cre2* derived cKO mice recapitulated the dentin and enamel phenotypes of *Din*-KO mice (Fig. 2A–C’). These results indicate that *Din* plays a critical role in dentinogenesis but is not essential for amelogenesis in murine incisors. The enamel defects observed in the incisors of *Din*-KO mice appear to be secondary effects resulting from defective dentinogenesis.

### Din is Essent ial for MSCs but not Odontoblasts in Murine Incisors.

To determine the cell stages at which *Din* functions in dental mesenchyme-derived cells, we conditionally knocked out *Din* from cells expressing *Col1a* and MSCs expressing *Gli1* using 2.3-kb *Col1a1-Cre* and *Gli1-Cre*^*ERT2*^. Surprisingly, *Din*-cKO from *Col1a*-expressing cells (odontoblasts and fibroblasts) did not result in any dental abnormities (Fig. 2D and D’), while *Din*-cKO from *Gli1*+ MSCs recapitulated a milder version of dentin and enamel defects similar to the *Din*-KO mice (Fig. 2E–G). These results collectively suggest that *Din* is dispensable for odontoblasts but essential for dental MSCs in murine incisors.

**Figure 3:**
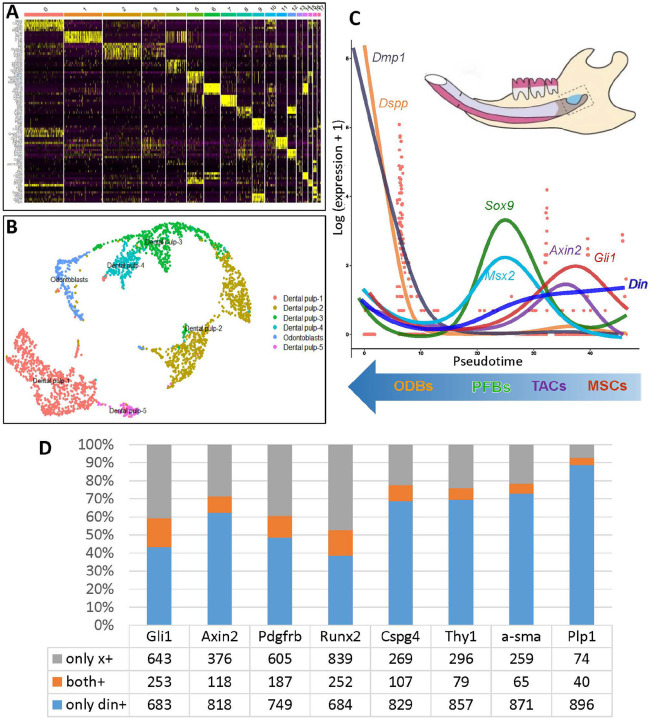
scRNA-seq analysis of incisor proximal tissues from 15-day-old WT mice. **A.** The cells in incisor proximal tissues were clustered into 17 groups based on their gene expression profile. **B.** Six clusters of dental pulp fibroblast cells excluding epithelial cells, blood cells, neuron cells, and immune cells, etc. **C.** Pseudotime analysis of pulp cell clusters determined the position of cells along the differentiation trajectory. Each peak expression of genes, including *Gli1, Axin2, Sox9, Msx2, Dspp*, and *Dmp1*, corresponded to a specific differentiation stage of cells: MSCs (mesenchymal stem cells), TACs (transit-amplifying cells), PFBs (pulp fibroblasts), and ODBs (odontoblasts), distinguished by different colors. The expression level of *Din* showed a gradually decreasing trend along the differentiation trajectory, peaking in MSCs and TACs, and declining to lower levels in pulp fibroblasts and odontoblasts. **D.** *Din* expression was detected in a fraction of MSCs in multiple MSC subpopulations.

In addition to *Gli1*+ MSCs, there are multiple MSC subsets in mouse incisors responsible for tissue homeostasis and/or injury healing^38^. To determine *Din* expression in the cell populations in murine incisors, we performed scRNA-seq on the proximal tissues from the lower incisors of P15 WT mice. Out of the 17 cell groups identified by cell clustering (Fig. 3A), we focused on the 6 groups from mesenchyme, including dental pulp, dental follicle, and odontoblasts (Fig. 3B). Pseudo-time assay revealed a decline in *Din* expression levels along the trajectory of MSC differentiation, with highest levels in MSCs and TACs, followed by low levels in pulp fibroblasts (PFbs) and odontoblasts (ODbs) (Fig. 3C). The scRNA-seq data further demonstrated *Din* expression in multiple MSC subsets in murine incisors, but in each subset, only a fraction of MSCs express *Din* (Fig. 3D). These data collectively suggests that *Din* is essential for dental MSCs but dispensable for odontoblasts. The *Din*-KO dental defects may involve multiple MSC subsets.

### Din is Essential for the Homeostasis of Dental MSCs During Postnatal Growth of Murine Incisors.

**Figure 4:**
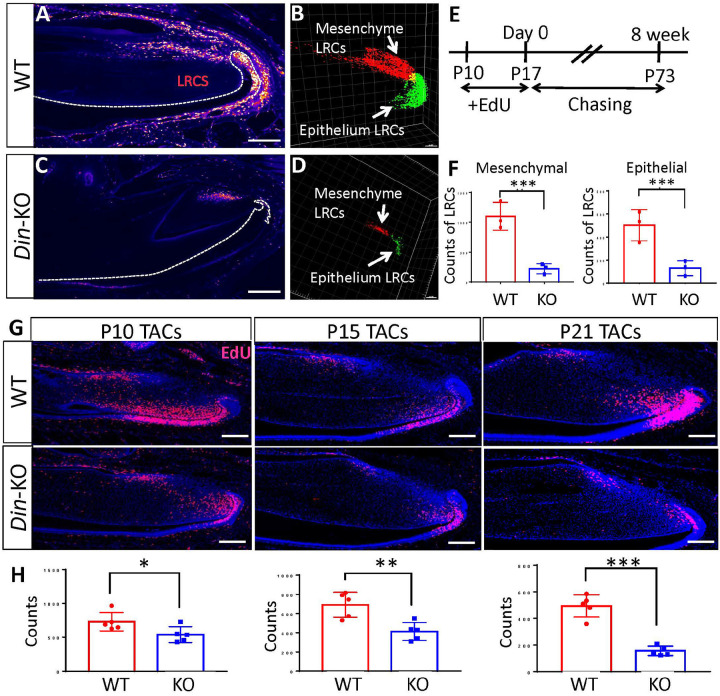
Label Retaining Cells (LRCs) and Transit Amplifying Cells (TACs) were significantly reduced in *Din*-KO incisors. **A.** An optical section of WT lower incisor showing LRCs in the stem cell niches. **B.** A 3D image reconstruction of epithelial and mesenchymal LRCs in WT incisors. **C.** An optical section of *Din*-KO lower incisor showing LRCs in the stem cell niches. **D.** A 3D image reconstruction of epithelial and mesenchymal LRCs in *Din*-KO incisors. **E.** EdU injection protocol for labeling LRCs. Mice were intraperitoneally injected with EdU, starting at P10 for 7 consecutive days. Mandibles were harvested after 8 weeks, on postnatal day 73. **F.** Statistic counts of dental epithelial and mesenchymal LRCs showed a significant reduction in *Din*-KO incisors. **G.** EdU assays indicated the proliferating TACs in lower incisors at P10, P15 and P21. **H.**The number of proliferating TACs in *Din*-KO incisors was significantly lower than in WT, and this difference worsened over time. N=3. *, P<0.05. **, P<0.01. ***, P<0.001. Scale bars, 200 μm.

Mouse incisor is an excellent model for studying homeostasis of stem cells due to its rapid turnover during postnatal growth and regenerative capacity. To determine the impact of *Din* deficiency on the homeostasis of dental MSCs, we examined the label retaining cells (LRCs) in mouse incisors. *Din*-KO and WT mice were i.p. injected with EdU (50 mg/kg) at P10 for 7 consecutive days, and sacrificed after 8 weeks. The mandibles were processed for tissue clearing and whole-mount EdU staining followed by confocal microscopy and Imaris quantitation as previously described^39, 40^. The *Din*-KO incisors showed dramatically less LRCs in both MSC niche and cervical loops than WT (Fig. 4A–D, F). Next, we examined TACs proliferation in incisor at P10, P15 and P21. EdU (50 mg/kg) was i.p. injected 2 h prior to sacrifice. IF staining of DSPP and AMEL was conducted on sagittal cryosections of lower incisors. *Din*-KO incisors showed reduced TACs in dental mesenchyme and epithelium, starting at P10 and worsening with age (Fig. 4G and H). Despite UniProt and MGI gene annotations associating *Din* with “cell apoptosis,” we did not observe significant changes in TUNEL staining in *Din*-KO incisors. (Fig. S3).

**Figure 5:**
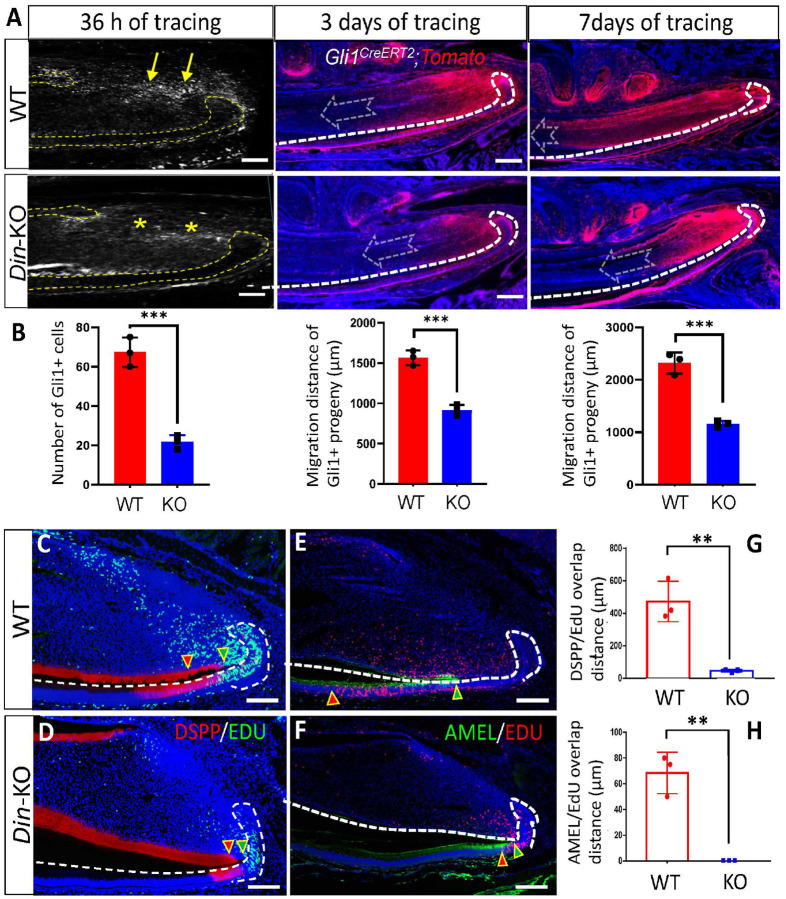
MSCs in *Din*-KO incisors showed impaired contribution to tissue turnover and differentiation potential. **A** and **B.** Lineage tracing of *Gli1*+ MSCs in lower incisors. After 36 h of lineage tracing, the *Din*-KO incisors showed reduced number of *Gli1*+ MSCs in the proximal stem cell niche (yellow asterisks and arrows). Following 3 and 7 days of lineage tracing, the *Din*-KO incisors exhibited compromised contribution of *Gli1*+ MSC progenies to tissue turnover toward the incisal side. **C, D** and **G.** Immunofluorescence staining of DSPP in *Din*-KO incisors revealed a strong expression on the proximal side near cervical loops compared to WT incisors. The overlap between DSPP and EdU (indicated by red and green arrowheads) was significantly diminished in *Din*-KO incisors, suggesting an impaired differentiation capacity of TACs. **E, F**and **H.** Immunofluorescence staining of AMEL in *Din*-KO incisors showed expression extending to cervical loops compared to WT incisors. The overlap between DSPP and EdU (indicated by red and green arrowheads) was significantly reduced in *Din*-KO incisors, indicative of a compromised differentiation potential of TACs. N=3. *, P<0.05. **, P<0.01. ***, P<0.001. Scale bars, 100 μm.

To determine the impacts of Din deficiency on the homeostasis of MSC progenies in mouse incisors, we performed lineage tracing of *Gli1*+ MSCs. *Gli1-CreERT2* and *tdTomato* transgenic mice were crossbred with *Din*-KO and WT mice. CreERT2 was induced by a single injection of tamoxifen (75 mg/kg, i.p.) at P11. The expression of tdtomato was traced for 36 h, 3 days, and 7 days. The *Din*-KO incisors showed significantly less *Gli1*+ MSCs than WT at 36 h, and their progeny cells displayed dramatically less/slower contribution to the tissue turnover from proximal toward incisal side at 3 and 7 days of tracing (Fig. 5A and B). IF staining detected robust expression of DSPP and AMEL on the proximal side near cervical loops in *Din*-KO incisors (Fig. 5C–F). The overlapping distance of DSPP/EdU or AMEL/EdU, indicative of the differentiation potential of TACs, was dramatically diminished in *Din*-KO incisors (Fig. 5C, E and G).These data strongly suggest that *Din* is essential for MSCs homeostasis in murine incisors during the postnatal growth and tissue turnover.

### Din is Essential for the Homeostasis of Dental MSCs During Injury Healing/Regeneration of Murine Incisors.

**Figure 6:**
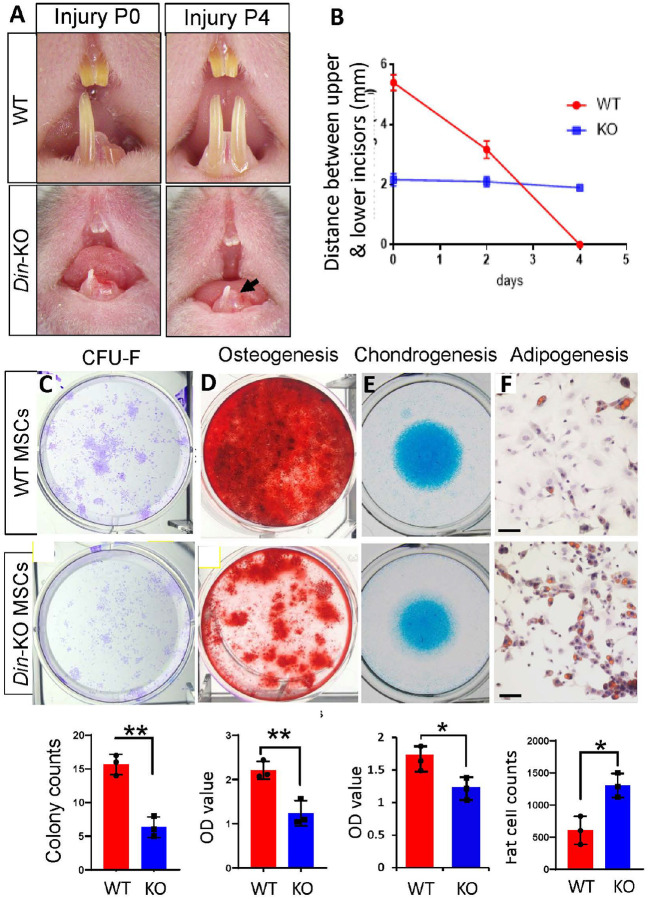
Injury healing, CFU-F and tri-lineage differentiation assays. **A** and **B.** Injury healing assay on lower incisors. The left lower incisors were clipped by removing the erupted part aligned with the gingival papilla. The fractured incisors of WT mice regrew to their normal length within 5 days. In comparison, the *Din*-KO incisors exhibited no discernible signs of healing growth or regeneration. **C.**CFU-F assay of MSCs isolated from lower incisors revealed significantly fewer colony formation by *Din*-KO MSCs compared to WT MSCs. **D** and **E.** MSCs isolated from *Din*-KO incisors showed compromised osteogenesis and chondrogenesis capacity compared to WT. **F.** *Din*-deficient MSCs showed enhanced adipogenesis capacity compared to WT. N=3. *, P<0.05. **, P<0.01. Scale bars in F, 100 μm.

The MSCs responsible for tissue homeostasis and injury repair are often attributed to different subpopulations^3, 11, 21, 38^. Our scRNA-seq and open-access data showed *Din* expression in multiple MSC subsets in murine incisors (Fig. 3D). To determine if Din is essential for the MSCs subsets responsible for injury healing, we conducted injury repair experiments on the lower incisors in 6-week-old mice^7, 41^. The broken incisors of WT mice regenerated to normal length in 5 days, while *Din*-KO incisors lacked regenerative healing (Fig. 6A and B). This data suggests that *Din* is essential for the homeostasis of MSC subsets responsible for injury healing and regeneration in murine teeth.

### Din Oversees Multiple Aspects of MSCs Homeostasis.

**Figure 7:**
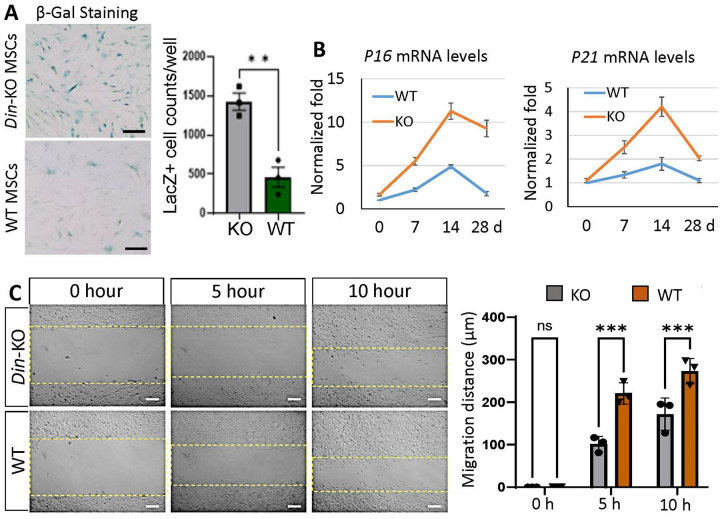
Senescence and motility assays. **A.** β-Gal staining showed significantly increased senescence in *Din*-KO MSCs than WT. n=3, **, P<0.01. **B.** Q-PCR analysis revealed elevated expression levels of *p16* and *p21* in *Din*-KO incisors from postnatal day 0 to day 28 compared to WT. **C.** Cell scratch motility assay demonstrated a significant reduction in motility of *Din*-KO MSCs compared to WT at 5 and 10 hours after scratching. N=3. ***, P<0.001. Scale bars, 100 μm.

To investigate whether *Din*-KO MSCs undergo cellular behavior/capability changes during tissue turnover and injury repair, we isolated *Gli1*+ MSCs from mouse incisors for CFU-F and tri-lineage differentiation assays. The *Din*-KO MSCs exhibited reduced capacities for CFU-F, osteogenesis, and chondrogenesis compared to normal controls (Fig. 6C–E), whereas their adipogenesis potential was significantly enhanced (Fig. 6F). Senescence assays, including β-Gal staining and Q-PCR analysis of senescence markers, unveiled a notable increase in aging cells and elevated expression levels of *P16* and *P21* in *Din*-KO MSCs (Fig. 7A and B). Scratch assays conducted on monolayer MSCs demonstrated a markedly impaired motility in *Din*-KO MSCs compared to normal controls. (Fig. 7C).

### Din Regulate MSCs Homeostasis through Rho GTPases.

**Figure 8:**
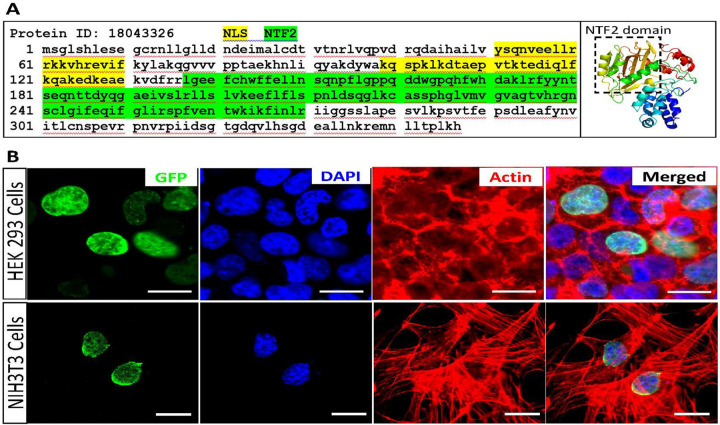
DIN protein is localized in cell nucleus (membrane). **A.** Amino acid sequence and predicted 3D structure of DIN protein. Left panel, bioinformatic analysis of DIN protein sequence revealed the presence of two Nuclear Localization Signals (NLS) and one Nuclear Transport Factor 2 (NTF2) domain within the DIN protein. Right panel, predicted 3D structure of DIN protein. NTF2 structure (boxed area) in the predicted DIN structure matches the reported NTF2 crystal structure. **B.** Subcellular location of DIN in cell nucleus. Transfection of *pCMV6-Din-GFP* expression vector into HEK-293 and NIH/3T3 cells showed robust localization of DIN-GFP fusion proteins in cell nucleus (membrane). Scale bars, 10 μm.

Using Phyre2 and cNLS Mapper analyses, we identified a Nuclear Transport Factor 2 (NTF2) domain and two Nuclear Localization Sequences (NLS) in the DIN protein. (Fig. 8A). NTF2 is a cytosolic protein mediating the import of GDP-bound small GTPase RAN (RAs-related Nuclear protein) from the cytoplasm into the nucleus, which is essential for the function of RAN in cargo receptor-mediated nucleocytoplasmic transport. The presence of NTF2 and NLS domains in DIN suggests that DIN may translocate into the cell nucleus to carry out its function. We determined the subcellular location of DIN by constructing and transfecting a *pCMV6-Din-GFP* mammalian expression vector into HEK-293 and NIH/3T3 cells. The DIN-GFP fusion proteins showed robust nuclear (membrane) localization in both cell lines (Fig. 8B).

**Figure 9:**
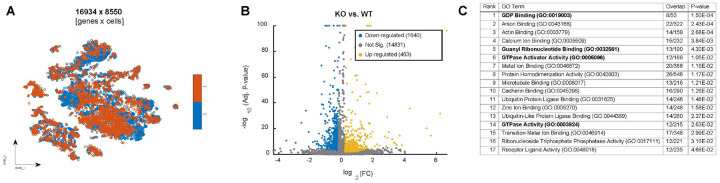
Transcriptomic alterations and pathway enrichment analysis between *Din*-KO and WT incisor fibroblasts revealed by scRNA-seq. **A.** tSNE projection of single cells colored by groups: WT orange and *Din*-KO blue. **B.** Volcano plot of differential gene expression between KO and WT cells. Each point represents a gene; significantly downregulated genes in KO are shown in blue (n = 1,640), upregulated genes in gold (n = 463), and non-significant genes in gray (n = 14,831). **C.** Result of enrichment analysis of downregulated genes using Enrichr. The enriched terms are shown, ranked by adjusted p-value, highlighting molecular functions associated with GTPase activity. “Overlap” indicates the number of DE genes found in each term relative to the total number of genes annotated to that term.

To investigate the potential mechanism through which *Din* regulates the homeostasis of dental MSCs, we compared gene expression in fibroblasts between *Din*-KO and WT incisors. The processed scRNA-seq data contains 16,934 genes expression in 8,550 fibroblast cells: 4,478 from the *Din*-KO samples and 4,072 from the WT samples (Fig. 9A). Using differential expression analysis, we identified 1,640 down-regulated genes (with expression level is lower in KO samples) and 463 up-regulated genes (with expression level is higher in KO samples, Fig. 9B). GO enrichment analysis with down-regulated genes identified 17 significant molecular function terms with Enrichr p-value < 0.05 (Fig. 9C). These include GDP Binding (GO:0019003), Guanyl Ribonucleotide Binding (GO:0032561), GTPase Activator Activity (GO:0005096), and GTPase Activity (GO:0003924). These results are consistent with the anticipated function of the NTF2 domain. Associated GTPase-related genes include: *Vav3, Arhgap10, Arhgap42, Acap2, Rgs3, Dlc1, Chn1, Arhgap29, Arhgap28, Iqgap2, Dnm1l, and Smap1*, which were all significantly downregulated in the *Din*-KO fibroblast populations (Supplementary Table 1).

To identify small GTPases that potentially interact with DIN, we conducted Co-IP assays on lysates prepared from NIH/3T3 cells stably transfected with *pCMV6-Din-HA* constructs, followed by LC-MS (liquid chromatography - mass spectrometry) assays (Fig. S4). Four Rho GTPases, namely Nras, RhoA, Cdc42, and Rac1, were identified as potential interactors with DIN, as evidenced by their 3.4 – 82.5 fold higher abundance in the DIN-HA pulled-down immunoprecipitations (Supplementary Table 2). The activity of these GTPases was further examined in the *Gli1*+ MSCs isolated from murine incisors using the RhoA/Rac1/CDC42 G-LISA Activation Assay Bundle Kit and Ras G-LISA Activation Assay Kit. The *Din*-KO MSCs showed significantly lower activity (GTP-bound) levels of RhoA and Rac1 compared to WT MSCs (Fig. S5), without apparent alteration in the levels of active Nras and Cdc42. These results collectively suggest that Din may positively regulate RhoA and Rac1 in MSCs within murine incisors.

## DISCUSSION

*Din* exhibits a broad expression pattern in both dental epithelial and mesenchymal naïve and differentiated cells. Correspondingly, *Din*-KO mice show defects in both dentin and enamel in their incisors. However, our lineage- and tissue-specific knockout studies targeting the epithelium, cranial neural crest, *Col1a1*-expressing cells, and *Gli1*+ MSCs demonstrated that *Din* is dispensable for odontoblasts and dental epithelium-derived cells (such as ameloblasts and ESCs) but is essential for dental MSCs in murine incisors. These findings indicate that the enamel defects observed in *Din*-KO mice are secondary to impaired dentinogenesis, and the primary dentin defects arise from abnormalities in MSCs but not from differentiated odontoblasts. Tooth development relies on proper epithelium-mesenchyme interactions. The epithelial or mesenchymal signaling can regulate the behavior of stem cells located in the counterpart compartment in murine incisors^6, 9, 42–45^. Thus, primary defects in the MSCs may have secondary effects on the ESCs and vice versa. In this study, both ESCs and MSCs in the *Din*-KO incisors exhibited a significant decrease in quantity, along with compromised proliferation and differentiation, suggesting that the potential interaction between MSCs and ESCs may be associated with the secondary enamel defects observed in the incisors of *Din*-KO mice.

Proper homeostasis of MSCs is crucial for the tissue turnover and injury repair in teeth^38^. Several studies have identified regulatory mechanisms governing certain aspects of MSC homeostasis in murine incisors^3–5, 7, 12, 20–22^. It remains unclear whether a cohesive regulatory mechanism oversees the homeostasis of MSCs. Through *in vivo* lineage tracing of MSCs in *Din*-KO and *Din*-cKO mice, along with *in vitro* characterization of the cellular potential and capacities of *Din*-deficient MSCs, we found that *Din* is essential for multiple aspects of MSC homeostasis, including maintaining stemness, migration, aging, proliferation, and differentiation. It plays a central role in regulating MSC homeostasis in murine incisors. This overarching effect is evident in two aspects: first, *Din* is expressed in multiple MSC subpopulations within murine incisors; second, *Din* may regulate MSCs through GTPases, which are binary molecular switches involved in the extensive regulation of various cellular processes. Through scRNA-seq and computational analysis, we identified a connection between Din and GTPases. Additionally, using IP, MS, and GLISA, we preliminarily identified several Rho GTPases that may interact with Din. Future studies will involve a series of *in vivo* and *in vitro* experiments to validate and further investigate the regulatory relationship between these molecules.

The expression of odontoblast and ameloblast differentiation markers, DSPP and AMEL, in the *Din*-KO incisors exhibited a ‘stronger’ and ‘more proximal’ pattern near the cervical loops, with reduced or absent overlap with EdU+ cells. It should be noted that this seemingly ‘stronger’ and ‘earlier differentiation’ pattern does not indicate enhanced proliferation and differentiation. On the contrary, it suggests a reduced capacity of the MSCs and ESCs regarding these aspects. The *Din*-deficient dental stem cells cannot supply adequate new TACs to support the turnover of ameloblasts and odontoblasts toward incisal side. Consequently, pre-odontoblasts and pre-ameloblasts differentiate into high-column mature cells in situ near the cervical loops, leading to robust DSPP and AMEL expression and the formation of mature enamel and dentin on the proximal side of the incisors.

The various MSC subpopulations in the dental pulp enable continuous growth and rapid repair of murine incisors. However, tissue turnover and injury repair are usually associated with different MSC subsets^3, 11, 21, 38^.For instance, *Gli1*+, *Thy1*+, and *Plp1*+ cells are slow-cycling cells regulating incisor tissue turnover and/or injury healing. *αSMA*+ and *Cspg4*+ MSCs function as pericytes involved in tissue healing following injury, whereas *Pdgfrb*+ and *Axin2*+ cells represent TACs derived from MSCs re-entering the cell cycle. Our scRNA-seq data showed that *Din* is expressed in most of the MSC subsets, and in each subset, only a fraction of MSCs express *Din*. It remains unclear whether the expression or absence of *Din* within the same subset leads to differences in MSCs behavior and cell fate. Future single-cell analysis and cellular characterizations after sorting the *Din*+ and *Din*− populations within each MSC subset and subset-specific knockouts may provide more valuable insights to clarify these questions. As a starting point, we knocked out *Din* from the largest MSC subpopulation in mouse incisors, the *Gli1*+ MSCs. The resulting conditional knockout incisors displayed a milder phenotype compared to the conventional knockout, which may be partially attributed to the efficiency of Cre, but more likely reflects *Din*’s role in multiple MSC subsets. Specifically, the knockout of *Din* in a single MSC subpopulation cannot fully recapitulate the knockout phenotype.

In summary, we have identified that a predictive gene *Din* plays central role in regulating multiple aspects of MSC homeostasis in murine incisors, which is essential for their continuous growth and injury repair. The mechanism underlying the regulation of incisor MSC homeostasis is associated with small GTPases.

## MATERIALS & METHODS

### Animals

All animal experiments were carried out according to the protocol approved by the Institutional Animal Care and Use Committee (IACUC) of Texas A&M University School of Dentistry (Dallas, TX, USA), and performed by the NIH Guide for the Care and Use of Laboratory Animals.

#### Generation of Din-KO^1st^, Din-KO and Din-flox Alleles

*Din*-KO^1st^ mice were generated using a gene-trap targeted ES cell clone *4930453N24Rik*^*tm1a(KOMP)Mbp*^ from The Knockout Mouse Project (KOMP). The *KO*^*1st*^ allele (Fig. 1A, tm1a) having *loxP* and *Frt* sequences can be transformed into conventional *Din-KO* (Fig. 1A, tm1b) or *Din*^*flox*^ (Fig. 1A, tm1c) allele via breeding with germline expressing *Cre* or *Flp* transgenic mice.

#### Generation of Din Conditional Knockout (cKO) Mice

All Cre lines used for generating cKO alleles were purchased from Jackson Laboratory (Bar Harbor, ME, USA). *Din-flox* mice were crossbred with *K14-Cre* (stock #004782), *Wnt1-Cre2* (stock #022501), *2.3Kb Col1a1-CreER*^*T2*^ (stock #016241) and *Gli1-CreER*^*T2*^ (stock #007913) mice to generate *K14*^*Cre/+*^;*Din*^*flox/flox*^*, Wnt1*^*Cre2/+*^;*Din*^*flox/flox*^*, 2.3KbCol1a1*^*CreERT2/+*^;*Din*^*flox/flox*^*, and Gli1*^*CreERT2/+*^;*Din*^*flox/flox*^ cKO mice, respectively. To induce CreER^T2^ expression, tamoxifen was administrated to mice by i.p. injection (75 mg/kg) at postnatal 5 days (P5) for 3 days.

### X-ray and Micro-CT

The dissected mandibles from 6-week-old mice were analyzed using X-ray radiography (Faxitron MX-20DC12, Tucson, AZ, USA). Micro-CT analyses were performed using a Scanco micro-CT35 imaging system (Scanco Medical, Bassersdorf, Switzerland) with a medium-resolution scan (7.0 μm slice increment) on mandibles. The.isq files obtained were read with KHKS-Scanco-ISQ-File Reader plugins installed in ImageJ. The.isq files were converted to.ids files using ImageJ. The.ids file obtained were converted into.ims file using Imaris File Converter. The.ims file was opened in the imaris Bitplane 9.01. Gross and sectional μ-CT imaging were obtained with pseudocolor rendering using the Imaris software.

### Histology

#### FFPE Tissue Sections

P15 and 6 weeks-old mice were harvested and mandibles were dissected and fixed with 4% paraformaldehyde (PFA) (Sigma Aldrich Corporation, St. Louis, MO, USA) in 0.1 M phosphate buffer solution (PBS) for 48 hours and then decalcified in 10% EDTA/PBS (pH 7.4) (Sigma Aldrich Corporation, St. Louis, MO, USA) at 4°C for 2 weeks. The tissues were processed for paraffin embedding, and 5 μm sections were prepared for hematoxylin & eosin (H&E) staining and other histological analyses.

#### RNAscope Staining of Gli1 and Din

Dual RNAscope staining was carried out with the RNAScope 2.5 HD Brown Duplex Reagent Kit (Advanced Cell Diagnostics, 322500, Newark, CA, USA) on 5-μm FFPE tissue sections prepared from mandibles of P15 mice according to manufacturer’s instructions. Briefly, slides were baked for 1 hour at 60 °C before use. After deparaffinization and dehydration, the tissues were air-dried and treated with a hydrogen peroxidase blocker before boiling at 100–104 °C in target retrieval reagents for 15 min. Protease was then applied for 30 min at 40 °C. Target probes LacZ and Gli1 (Advanced Cell Diagnostics, 461191, 311001) were hybridized for 2 h at 40 °C, followed by a series of signal amplification and washing steps. All hybridizations at 40 °C were performed in HybEZ^™^ II Hybridization System (Advanced Cell Diagnostics). RNA staining signal was identified by two colors: green chromogenic dots developed by HRP for the *Gli1* probe and red chromogenic dots developed by AP for the *LacZ* probe. Following the RNAscope assay, samples were counterstained for 2 min with hematoxylin.

#### Cryosection and X-galactosidase (Gal) Staining

The *Din-KO* allele has a LacZ reporter driven by endogenous *Din* promoter, which can be used for indicating *Din* expression (Fig. 1A. tm1b). X-Gal Staining was performed on the cryosections of mandibles obtained from 4 weeks old *Din-KO* heterozygous (normal) mice as described previously^39, 46^. Briefly, tissues for cryosection were fixed in 4% PFA for 24 hours at 4 °C followed by decalcification in 10% EDTA for 2 weeks. 30% sucrose dehydration at 4 °C overnight followed by OCT embedding for cryosection. Cryo-sections were fixed with 0.2% glutaraldehyde in PBS at 4 °C for 30 min. The sections were washed for three times in 0.005% NP-40 and 0.01% sodium deoxycholate and then incubated in staining solution (5 mM potassium ferrocyanide and potassium ferricyanide, 2 mM MgCl2, 0.4% X-Gal in dimethylformamide) at 37 °C for 3–24 hours. Post fixation was done with 4% PFA in PBS at room temperature for 1 hour followed by counterstaining with nuclear fast red (Vector Laboratories Inc, Newark, CA, USA)

#### Cell Proliferation (EdU) and TUNEL Assays

Mice were i.p. injected with EdU (50 mg/kg in PBS, ThermoFisher, C10352, Carlsbad, CA, USA). The mandibles were collected 2 h after injection and processed for cryosection or FFPE section. EdU incorporation was detected using a Click-iT Kit (ThermoFisher Scientific, C10337, Waltham, MA, USA) as we previously described^39^. For TACs assay, mice were injected EdU at P10, P15 and P21 days.

Apoptotic cells were examined on the FFPE sections via TUNEL staining using an ApopTag Plus In Situ Apoptosis Fluorescein Detection Kit (Millipore, S7111, Burlington, MA, USA) according to the manufacturer’s instruction. DAPI was used as counterstaining.

#### Immunofluorescece Staining of DSPP and AMEL with EdU

EdU injection, mandible collection, paraffin embedding, FFPE section and IF staining were performed as described above. IF staining of DSPP and AMEL was performed as previously described^40^. The primary antibodies were anti-DSPP (1:200, Abcam, ab216892, Burlingame, CA, USA) and anti-AMEL (1:500, Santa Cruz Biotechnology, sc-32892, Dallas, TX, USA). Primary antibodies were detected using AlexaFluor488 conjugated secondary antibody and AlexaFluor633 conjugated secondary antibody (ThermoFisher, A-11034, and A-21070). DAPI was used for counterstaining.

### Label Retaining Cell Assay

*Din*-KO and WT mice were i.p. injected EdU (50 mg/kg) at P10 for consecutive 7 days, and sacrificed at postnatal 8 weeks as described previously^47^. Briefly, the mandibles were fixed with 4% PFA followed by decalcification and PEGASOS tissue clearing^48^. Fluorescent imaging was acquired by an SP8 Confocal Microscope (Leica Microsystems, Wetzlar, Germany). Image processing and 3D rendering were performed with Imaris 9.0 (Bitplane, Belfast, UK) as described previously^47^.

### Single-Cell RNA Sequencing (scRNA-seq)

#### Preparation of Single Cell Suspension

The proximal tissues of the lower incisors of P15 *Din*-KO mice and WT mice were obtained with fine forceps in ice-cold PBS. The tissue was minced into 0.5 mm pieces and dissociated into single-cell suspension by digesting in a-MEM containing 2.5 mg/ml liberase and 1U/μl DNase at 37C° for 30 min, then terminated with 10% FBS and passed through Pluri-strainer Mini 70μm (PluriSelect, 43-10070-40, El Cajon, CA, USA). Dead cells were removed using the Dead Cell Removal Kit (Miltenyi Biotec, 130-090-101, MD, USA).

#### Library Construction and Sequencing

Cell barcoding, cDNA synthesis, and library preparation were done following the protocols of 10X Next GEM single-cell 3’ reagent kits V3.1 (10× Genomics, 1000128, Peasanton, CA, USA). Quality control for cDNA and library was performed on a 2100 Bioanalyzer (Agilent Technology). Sequencing was performed with NovaSeq PE-150 at LC Sciences (Houston, TX, USA).

#### Bioinformatics Analysis of scRNAseq Data

Sequencing results were demultiplexed and converted to FASTQ format using Illumina bcl2fastq software. The CellRanger (https://support.10xgenomics.com/single-cell-gene-expression/software/pipelines/latest/what-is-cell-ranger) was used to perform barcode processing and 3’ gene counting. The cDNA insert was aligned to the mm10/GRCm38 reference genome. Only confidently mapped, non-PCR duplicates with valid barcodes and UMIs were used to generate the gene-barcode matrix. Further analysis included quality filtering, the identification of highly variable genes, dimensionality reduction, and standard unsupervised clustering. Low-quality cells with fewer than 1,000 detected genes were removed. Cells with more than 10% of the transcripts coming from mitochondrial genes or more than 3% Hbb/Hba content were also removed. Dimensionality reduction was performed with UMAP as described previously^49^. A joint analysis of *Din*-KO and WT data was performed following the Seurat integration procedure^50, 51^. Cell-cycle scoring and regression were performed with the default list of cell-cycle genes. Clustering was performed with ‘FindClusters’ and ‘resolution=1’. Cluster marker genes were identified with ‘FindAllMarkers’. Trajectory analyses were carried out on the odontogenic subset using Monocle 3 and TradeSeq^52, 53^. Cell-cell communication analysis were performed using CellChatand CellphoneDB v2.1.4^54, 55^.

#### Re-visiting Open-Access scRNA-seq Data

The raw scRNA-seq data matrices of dental cell type atlas available online from PK lab, Harvard University, were analyzed through bioinformatics using the R codes for retrieving each cluster from the data matrix to examine the expression pattern of *Din* in different cell populations in mouse incisors. Dimensionality reduction and cluster annotation information of cell subgroups in the whole incisor was performed and the cell subgroups expressing *Din* were analyzed tSNE embedding and cluster annotation information was obtained at: http://pklab.med.harvard.edu/ruslan/dental.atlas.html. R codes for retrieving each cluster from the matrix were obtained at: http://pklab.med.harvard.edu/ruslan/tooth_atlas/scripts/. The data analysis protocol was followed as described previously^56^.

### Lineage Tracing of Gli1+ MSCs

*Gli1-CreER*^*T2*^ and *td*-Tomato mice were crossbred with *Din*-KO mice to generate *Gli1*^*CrERT2/+*^;*Din-KO*^*+/−*^;*td-Tomato* (heterozygous normal) and *Gli1*^*CrERT2/+*^;*Din-KO*^*−/−*^;*td-Tomato* (homozygous KO) mice. CreER expressions were induced by a single injection of tamoxifen (75 mg/kg, i.p.) at P11, and td-Tomato expressions were traced for 36 h, 3 days and 7 days. The tissues were harvested and processed for cryosections. 10 μm cryosections were prepared in cryotome (Leica, CM 1860) and processed for fluorescent microscopy using a scanning microscope (Olympus, BX61VS Slide Scanners, Center Valley, PA, USA).

### Incisor Injury Repair Assay

6-weeks old *Din*-KO mice and their heterozygous (normal) littermates were used for injury repair assays. The mice were anesthetized with isoflurane. The left lower incisors were clipped at the level of gingival papilla by removing the erupted part. The length of broken incisors was measured with a digital caliper (VWR, Radnor, PA, USA) on daily basis for 10 days.

### Primary Culture of Dental Pulp Gli1+ MSCs and CFU-F Assay

Incisor pulp was obtained from 6 weeks-old *Gli1*^*CrERT2/+*^;*Din-KO*^*+/−*^;*td-Tomato* (heterozygous normal) and *Gli1*^*CrERT2/+*^;*Din-KO*^*−/−*^;*td-Tomato* (homozygous KO) mice after tamoxifen induction for 2 days. The dental epithelium was carefully removed with fine forceps.The pulp tissue was minced into 0.5 mm pieces and dissociated into single-cell suspension by digesting in a-MEM containing 2.5 mg/ml liberase and 1U/μl DNase at 37C° for 30 min, then terminated with 10% FBS and passed through Pluri-strainer Mini 70μm (PluriSelect). The cell suspension was sorted using the FACSAria II (BD Biosciences, Franklin Lakes, NJ, United States).

For CFU-F (colony forming unit-fibroblasts) assay, the *Gli1*+ MSCs obtained from cell sorting were seeded in a 100 mm plate at approximately 1million cells per 10 ml of growth medium. The cells were grown for 3 days and the growth medium changed to remove unattached cells and selectively grow the adherent MSCs. The adherent MSCs were further grown until the 5^th^ day to reach approximately 70% confluency. Passage 2 MSCs were seeded in 6 well plates at a low density of 1×10^4^ cells and grown in growth media containing a-MEM, 10% FBS, 1% antibiotic-antimycotic for 3 weeks. Cells were washed with PBS and fixed with 100% methanol. Cells were stained with crystal violet solution. Gross photographs of the stained plates were taken and the CFU-F colonies were counted manually.

### Tri-lineage Differentiation Assay

For osteogenesis assay, the *Gli1*+ MSCs obtained from cell sorting were cultured in 6 well plates at a density of 4.2 ×10^3^ cells/cm^2^ in 2 ml StemXVivo Osteogenic/Adipogenic Base Media (Bio-Techne, CCM007, Minneapolis, MN, USA) with 1% antibiotic-antimycotic (ThermoFisher) until 70 % confluency was reached. The cells were further grown in differentiation media with StemXVivo Mouse/Rat Osteogenic supplement (Bio-Techne, CCM009) for 3 weeks with media changed every 3 days. The cells were stained with Alizarin Red S and the photograph was obtained. The cells were scrapped with 10 % acetic acid, mixed homogenously in a vortex, and centrifuged, the colored supernatant was collected and neutralized with 10% ammonia water. The colorimetric assay was performed to obtain the OD values using a Biotek Cytagen Gen5 microplate reader (Agilent, Santa Clara, CA, USA) and the differences were shown in statistics.

For adipogenesis assay, the MSCs in StemXVivo Osteogenic/Adipogenic Base Media reached 100 % confluency were further grown in differentiation media made with Human/Mouse/Rat StemXVivo^®^ Adipogenic Supplement (Bio-Techne, CCM011) for one week with media changed every 3 days. The cells were stained with Sudan Red III and counterstained with Mayers Hematoxylin. The bright field imaging of cells was done under the inverted microscope. Random pictures of cells were taken from 10 different microscopic fields and the number of red-stained adipocytes were counted manually between *Din*-KO and WT cells.

For chondrogenesis assay, 1.25 × 10^5^ MSCs in StemXVivo Chondrogenic Base Media (Bio-Techne, CCM005) were centrifuged at 200 × g for 5 min to pellet the cells. The pellets in tubes were incubated upright in completed StemXVivo Chondrogenic Differentiation Media (Bio-Techne, CCM006) at 37° C and 5% CO_2_ for 2 days with media changed every 2–3 days. Chondrogenic pellets were harvested after 20 days in culture and imaged under the inverted microcope.

### Cell Scratch Motility Assay

*Din*-KO and WT MSCs were cultured in 6 well plates in StemXVivo Mesenchymal Stem Cell Expansion Media (Bio-Techne, CCM004) to obtain a cell monolayer of 70 % confluency. Plastic pipette tip was used to make a straight scratch line on cells monolayer. Cell migration towards the scratch line were observed in 0 , 5 and 10 h and microscopic photographs taken. The distance between two scratch lines in the photographs were measured in Image J software to determine differences in the cell migration distance between *Din*-KO and WT MSCs.

### Senescence β-Gal Staining

*Din*-KO and WT MSCs were cultured in 12 well plates in StemXVivo Mesenchymal Stem Cell Expansion Media to obtain a cell monolayer of 80 % confluency. The senescence of *Din*-KO and WT MSCs was examined by β-Gal Staining kit (Cell Signaling, 9860, Cell Signaling, Danvers, MA, USA) following the manufacturer’s instructions. Random pictures of cells were taken from 10 different microscopic fields and the number of blue-stained senescence cells were counted manually between *Din*-KO and WT cells.

### Q-PCR

The proximal tissues of lower incisors were dissected from postnatal 0, 7, 14, and 28-day-old *Din*-KO and WT mice using fine forceps under a stereo microscope. The tissues were flash-frozen in liquid nitrogen, crushed using a mortar and pestle, and total mRNA was extracted using a RNeasy Kit (QIAGEN, 74104, Germantown, MD, USA) following the manufacturer’s protocol. 1 μg total RNA was used to make cDNA using a Reverse Tanscriptase kit (QIAGEN, 220211). P16 and P21 qPCR primers were designed, and the mRNA levels were determined using quantitative real-time PCR (qRT-PCR) analysis performed in triplicates using the SYBR Green method in the Biorad CFX 100 qPCR analyzer. The housekeeping gene, *gapdh*, was used to normalize the expression levels of target genes.

### Microscopy

Bright-field images were acquired by an Olympus CX43 Upright Light Microscope or an Olympus CKX41 inverted microscope connected with a DP27 imaging system (Olympus). Immunofluorescent images were acquired by an Olympus BX61VS Slide Scanner (Olympus) or an SP8 confocal microscope (Leica).

### Mammalian Expression Vectors

#### pCMV6-Din-GFP vector:

The cDNA of mouse *Din* (*4930453N24Rik*) was synthesized by GenScript with HindIII and MluI restriction sites on 5’ and 3’ ends and subcloned into *pCMV6-AC-GFP* vector (OriGene, PS100010, Rockville, MD, USA) to form a *pCMV6-Din-GFP* vector for mammalian expression.

#### pCMV6-Din-HA Vector:

The *GFP* sequence in *pCMV6-Din-GFP* vector constructed above was removed by MluI and PmeI restriction enzymes. An *HA cDNA* sequence synthesized by GenScript with MluI and PmeI restriction sites on 5’ and 3’ was subcloned into the *GFP*-removed notch to form a *pCMV6-Din-HA* vector for mammalian expression.

### Transient and Stable Cell Transfection

For transient expression of Din-GFP fusion proteins, HEK293 and NIH/3T3 cells were seeded in a 12-well plates in 1×10^5^/cm^2^ density, and grown for 70% confluency in a growth medium containing α-MEM, 10% Heat Inactivated FBS and 1% antimycotic-antibiotic. The *pCMV6-Din-GFP* plasmid was transfected to the cells using Lipofectamine 3000 (ThermoFisher, L3000001) in Opti-MEM media (ThermoFisher, 31985070) following the manufacturer’s instructions. The cells were fixed with 10% neutral buffered formalin and permeabilized with 0.1% Triton-X 100 in PBS and immunostained with β-Actin primary antibody (Cell Signaling, 4967) and nuclei counterstained with Gold Antifade Reagent with DNA Stain DAPI (ThermoFisher, P36931). Imaging was done using an SP8 confocal microscope (Leica).

For stable expression of Din-HA fusion proteins, NIH/3T3 cells transfected with *pCMV6-Din-HA* plasmids were selected with neomysin following ThermoFisher Stable Transfection Protocol. The expression of DIN-HA fusion proteins was confirmed by Western Blotting using anti-HA antibody (ThermoFisher, 26183). Cells stably transfected with *pCMV6-AC-GFP* plasmids were used as blank control.

#### Co-Immunoprecipitation and Mass Spectrometry

Cells stably expressing *Din-HA* were harvested at 90% confluency and lysed on ice for 15 min. Co-IP was performed using Pierce^™^ HA-Tag IP/Co-IP Kit (ThermoFisher, 26180) according to the manufacturer’s instructions. The precipitated proteins with DIN-HA were eluted with SDS sample buffer and heated at 95C° for 10 min and subjected to BCA quantitation. Equal amounts of proteins from each group were loaded on SDS-PAGE. Coomassie Blue stained protein bands were cut off and diced into ~1 mm^3^ pieces, then digested overnight with trypsin followed by reduction and alkylation with DTT and iodoacetamide. After solid-phase extraction clean-up, 2 μl of each sample was examined on a QExactive HF mass spectrometer coupled to an Ultimate 3000 RSLCNano liquid chromatography system (LC-MS) (ThermoFisher).

The LC-MS data were analyzed with Proteome Discoverer v2.4 (ThermoFisher), with peptide identification performed using Sequest HT searching against the mouse protein database from UniProt. Fragment and precursor tolerances of 10 ppm and 0.02 Da were specified, and three missed cleavages were allowed. Carbamidomethylation of Cys was set as a fixed modification, with oxidation of Met set as a variable modification. The false-discovery rate cut-off was 1% for all peptides. Proteins that were identified with only 1 peptide-spectrum match, identified but not quantified, or with potential contaminants were excluded from further analysis. The downstream analysis followed the workflow of the DEP package^57^. Protein-wise linear models combined with empirical Bayes statistics were used for the differential expression analysis. Empty vector IPs were used as experimental controls to provide a background list of proteins binding non-specifically to the construct. Proteins were filtered for protein fold change equal to or greater than 2.0. GO enrichment of differentially expressed proteins was performed using the Clusterprofile R package.

### G-LISA Assays

The dental pulpMSCscells isolated above were subjected to serum starvation for 48 h to lower the basal levels of GTPase activity and increase the response to a given activator. The cells were then activated with a 0% FBS growth medium containing Rho/Rac/CDC42 Activator I (Cytoskeleton Inc., CN04-A, Denver, CO, USA) for 2.5 hours, and with EGF for 3 min (Cytoskeleton, CN02) for Ras GTPases activation. The cells without RhoA/Rac/CDC42 Activator I and EGF were used as a control to measure the basal GTPase levels. Immediately the cells were washed with 10 ml ice-cold Ca^+^ and Mg^++^ free DPBS and lysed in 600 μl ice-cold cell lysis buffer on ice following the manufacturer’s instructions. The cells were scraped and collected in prechilled 1.5ml EP tubes and centrifuged at 10,500 rpm at 4°C for 1 min. The supernatant obtained was immediately aliquoted in multiple prechilled 1.5 ml EP tubes on ice. The cell lysates aliquots were flash-frozen in liquid nitrogen and stored at −80°C. The protein concentration of each sample was measured as per the manufacturer’s instructions using the 600 nm wavelength of BioTek Cytation 5 Spectrophotometer (Agilent). Ras/RhoA/Rac1/CDC42 GLISA Assay was performed adhering to the manufacturer’s instructions and recommendations. Briefly, each protein sample concentration was equalized using the respective cell lysis buffer and subjected to Ras/RhoA/Rac1/CDC42 GLISA Assay. The final Ras/RhoA/Rac1/CDC42 activity was measured using the 490 nm wavelength of BioTek Cytation 5 Spectrophotometer comparing WT and *Din*-KO MSCs.

### Protein Domain Prediction

Phyre2 and cNLS Mapper were employed for protein domain prediction.

### Statistics

At least three independent samples were used in analysis wherever required and the data were expressed as mean ± SD. Unpaired Student’s t-test and one-way ANOVA were used to analyze data sets with two groups and when comparing more than two groups respectively. For one-way ANOVA, p-values were determined followed by Tukey’s multiple comparison tests. Prism software package (GraphPad Prism 9) or Microsoft Excel were used in statistical calculations. *P<0.05, **P<0.01, ***P< 0.001, and ****P<0.0001 were used to determine statistically significant differences.

## Supplementary Files

This is a list of supplementary files associated with this preprint. Click to download.

- FigS1.jpg
- FigS2.jpg
- FigS3.jpg
- FigS4.jpg
- FigS5.jpg
- supplementarytable1.xlsx
- supplementarytable2.xlsx

## Data Availability

The sequence data reported in this article have been deposited in the NCBI Sequence Read Archive (SRA) (BioProject ID: PRJNA1256476). The scRNA-seq expression matrices have been deposited in the NCBI Gene Expression Omnibus (GEO) database (GSE296021). The mass spectrometry data reported in this article have been deposited in MassIVE (ID: MSV000097647).
